# FLIM-FRET analyzer: open source software for automation of lifetime-based FRET analysis

**DOI:** 10.1186/s13029-017-0067-0

**Published:** 2017-11-03

**Authors:** Jiho Kim, Yury Tsoy, Jan Persson, Regis Grailhe

**Affiliations:** 1 Technology Development Platform, Institut Pasteur Korea, 463-400, 16, Daewangpangyo-ro 712 beon-gi,l Bundang-gu, Seongnam-si, Gyeonggi-do Republic of Korea; 2 Image Mining, Institut Pasteur Korea, 16, Daewangpangyo-ro 712 beon-gil, Bundang-gu, Seongnam-si, Gyeonggi-do 463-400 Republic of Korea

**Keywords:** Fluorescence lifetime imaging microscopy (FLIM), Fluorescence resonance energy transfer (FRET) analysis, Intensity-based segmentation, Batch processing

## Abstract

**Background:**

Despite the broad use of FRET techniques, available methods for analyzing protein-protein interaction are subject to high labor and lack of systematic analysis. We propose an open source software allowing the quantitative analysis of fluorescence lifetime imaging (FLIM) while integrating the steady-state fluorescence intensity information for protein-protein interaction studies.

**Findings:**

Our developed open source software is dedicated to fluorescence lifetime imaging microscopy (FLIM) data obtained from Becker & Hickl SPC-830. FLIM-FRET analyzer includes: a user-friendly interface enabling automated intensity-based segmentation into single cells, time-resolved fluorescence data fitting to lifetime value for each segmented objects, batch capability, and data representation with donor lifetime versus acceptor/donor intensity quantification as a measure of protein-protein interactions.

**Conclusions:**

The FLIM-FRET analyzer software is a flexible application for lifetime-based FRET analysis. The application, the C#. NET source code, and detailed documentation are freely available at the following URL: http://FLIM-analyzer.ip-korea.org.

**Electronic supplementary material:**

The online version of this article (10.1186/s13029-017-0067-0) contains supplementary material, which is available to authorized users.

## Introduction

Over the past decades, light microscopy has been customized to facilitate the investigation of protein assembly into macromolecular complexes in living cells. Calculating the efficiency of the Förster/fluorescence resonance energy transfer (FRET) allows characterization of protein-protein interactions or changes in protein conformation, because the efficiency varies as the inverse sixth power of distance between fluorophores, typically reaching 50% at 2–8 nm [1]. Among the few existing approaches toward detecting and quantifying FRET, fluorescent lifetime imaging (FLIM) has a number of advantages. The changes in fluorescence lifetime are independent of fluorophore concentration, simplifying the process of filtering out artifacts introduced by variations in fluorophore concentration and emission intensity across the sample. In contrast to intensity-based FRET measurements, FLIM measurements are relatively robust under conditions where spectral crosstalk is present. As a result, FLIM experiments do not require spectral calibration measurements. Fluorescence lifetime can also be used to distinguish different fluorophores with similar spectral properties [2] and report variations in the fluorophore’s local environment [3]. Although FLIM is widely used, it is subject to major limitations. A few softwares are available for batching the analysis of FLIM data [4–6], but none provide automated segmentation function for single cell lifetime analysis. Such limitations make FLIM data analysis extremely time intensive, which is a major drawback for protein-protein interaction studies. We therefore have developed a single cell image segmentation software called FLIM-FRET analyzer, which separates objects of interest from the background to delineate whole cells, facilitating image segmentation into single cells followed by donor lifetime and donor/acceptor fluorescence intensity quantification.

## Implementation

**Fig. 1:**
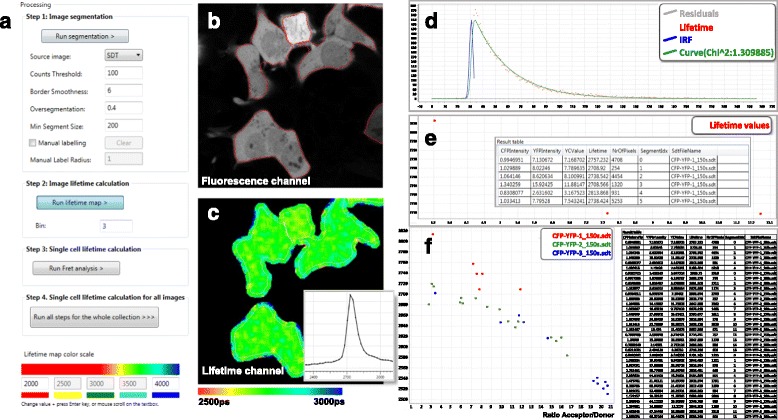
Illustration of the features of FLIM-FRET analyzer. **a** Multistep menu for single cell segmentation (step 1), whole image lifetime calculation (step 2), average lifetime single cell calculation (step 3), and batched multi FLIM image experiments (step 4). **b** Fluorescence-based (*white*) single cell segmentations (*red*). **c** Lifetime map. **d** Instrument response function (*blue)*, Lifetime raw data (*red*), Lifetime fitting (*green*). **e** Average lifetime calculation at the single cell level. **f** Batched analysis of multiple experiments and the average single cell FLIM measurement display

FLIM-FRET analyzer was designed to automate processing of intensity-based cell image segmentation, fluorescence lifetime fitting, and FRET analysis of entire data sets. FLIM-FRET analyzer was designed to automate processing of intensity-based cell image segmentation, fluorescence lifetime fitting, and FRET analysis of entire data sets. This software consists of two distinct processes: The first part is to create a FRET collection which associates fluorescent intensity and fluorescence decay image datasets. To create a FRET collection, the user imports the donor and acceptor fluorescent channels (.tiff files) and the donor fluorescence decay curves (.sdt files), which in our use-case were obtained with a Single Photon Counting module (SPC-830; Becker & Hickl GmbH). During step 1 of data analysis, the .sdt or .tiff file is selected, and the cell or organelles are segmented based on size and fluorescence intensity (Fig. 1a-b). After cell segmentation, the lifetime fitting is performed using the exponentially modified Gaussian curve [7]. During step 2, binning is set to select the optimal tradeoff between spatial resolution and the number of photons acquired per pixel. A color-coded lifetime image (red to blue) is displayed after the fitting process is complete. The lifetime calculation step produces two output tabs. The first tab (Fig. 1c, inset) contains the lifetime histogram, which illustrates the lifetime distribution for the selected images. The second tab (Fig. 1d) contains the fluorescence decay curve raw data and the fitted trace for the selected pixel (the quality of fit is determined by the chi-square value (χ^2^). Step 3 consists of the single cell lifetime calculation. Choosing “Run FRET analysis” enables the quantification of the acceptor/donor intensity ratio and the average lifetime for each segmented single cell. A plot of average lifetime versus acceptor/donor intensity for each single cell is automatically displayed, as seen Fig. 1e. This plot is very useful for visualization of the expected correlation between FRET and acceptor/donor intensities. It is also possible to launch all three described steps for all data within the stack by choosing “Run all steps for the whole collection”. The FRET analysis results appear as a pop-up window as shown in Fig. 1f. In batch analysis, step 4 involves summarizing the data in a table as well as presenting a scatterplot of lifetime versus the acceptor/donor intensity ratio.

## Results and discussion

We tested the performance of FLIM-FRET analyzer against that of the standard SPCImage (version 5.4) software, which requires manual segmentation of single cells, using HEK-293 cells expressing fluorescent proteins. Both methods yielded comparable results (Pearson r: 0.9925), suggesting that FLIM-FRET analyzer software is a robust and accurate tool for analyzing protein-protein interactions using fluorescence assays (Additional file 1: Figure S1). Last, we illustrate the application of FLIM-FRET Analyzer upon lifetime quantification of single cell expressing FRET-capable pairs of fluorescent proteins (Additional file 1: Figure S2).

## Conclusion

The stand-alone software described here aims to simplify and accelerate the process of analyzing multivariate FLIM data sets for single cell lifetime quantification. The available full C# source code (http://FLIM-analyzer.ip-korea.org.) will allow the user to adapt or extend the currently provided version of the application. A tutorial (Additionnal file 2) can also be downloaded at the same URL.

## Additional files

Additional file 1: Supplementary figures 1 & 2. **Figure S1.** Comparison between FLIM-FRET analyzer and SPCImage software. Lifetime imaging measurement of the Cerulean Cyan Fluorescent Protein linked with Yellow Fluorescent Protein (CFP-YFP) chimera expressed in HEK-293 cells, using the microscope LEICA TCS SP2 combined with SPC-830 module (Becker & Hickl GmbH). A. Representative lifetime image was automatically segmented (red line) into four segmented cells which were independently analyzed by FLIM-FRET analyzer. B. Multi step process to analyze the fluorescence lifetime and distribution for each of the four cells using by SPCImage software. (The ROI in SPCImage software was manually selected.) C. The lifetime values calculated using FLIM-FRET analyzer shows high correlation (Pearson *r* > 0.99) with the values obtained with the SPCImage software. We additionally found the lifetime values of FLIM-FRET analyzer to be slightly longer than of the SPCImage, by a factor of 1.17±0.03. **Figure S2.** Validation of the FLIM-FRET analyzer using negative and positive FRET control probes expressed in cells. A. Lifetime images of CFP, CFP plus YFP, and CFP-YFP expressing HEK-293 cells processed with the FLIM-FRET analyzer. B. Comparative analysis of the fluorescence lifetime of single cells expressing CFP, CFP plus YFP, and CFP-YFP shows significant drop of the fluorescence lifetime for the C-Y chimera known to FRET. (PPTX 2044 kb)

Additional file 2: User guide. (DOCX 4536 kb)
