# Supplementary material for: FLIM-FRET analyzer: open source software for automation of lifetime-based FRET analysis
Source: Source Code Biol Med. 2017 Nov 3;12:7. doi: 10.1186/s13029-017-0067-0 (PMC5670528; doi:10.1186/s13029-017-0067-0)
Supplement: Supplementary file 1 — Figure S1. Comparison between FLIM-FRET analyzer and SPCImage software. Lifetime imaging measurement of the Cerulean Cyan Fluorescent Protein linked with Yellow Fluorescent Protein (CFP-YFP) chimera expressed in HEK-293 cells, using the microscope LEICA TCS SP2 combined with SPC-830 module (Becker & Hickl GmbH). A. Representative lifetime image was automatically segmented (red line) into four segmented cells which were independently analyzed by FLIM-FRET analyzer. B. Multi step process to analyze the fluorescence lifetime and distribution for each of the four cells using by SPCImage software. (The ROI in SPCImage software was manually selected.) C. The lifetime values calculated using FLIM-FRET analyzer shows high correlation (Pearson r > 0.99) with the values obtained with the SPCImage software. We additionally found the lifetime values of FLIM-FRET analyzer to be slightly longer than of the SPCImage, by a factor of 1.17±0.03. Figure S2. Validation of the FLIM-FRET analyzer using negative and positive FRET control probes expressed in cells. A. Lifetime images of CFP, CFP plus YFP, and CFP-YFP expressing HEK-293 cells processed with the FLIM-FRET analyzer. B. Comparative analysis of the fluorescence lifetime of single cells expressing CFP, CFP plus YFP, and CFP-YFP shows significant drop of the fluorescence lifetime for the C-Y chimera known to FRET. (PPTX 2044 kb) [file 13029_2017_67_MOESM1_ESM.pptx]

## Slide 1
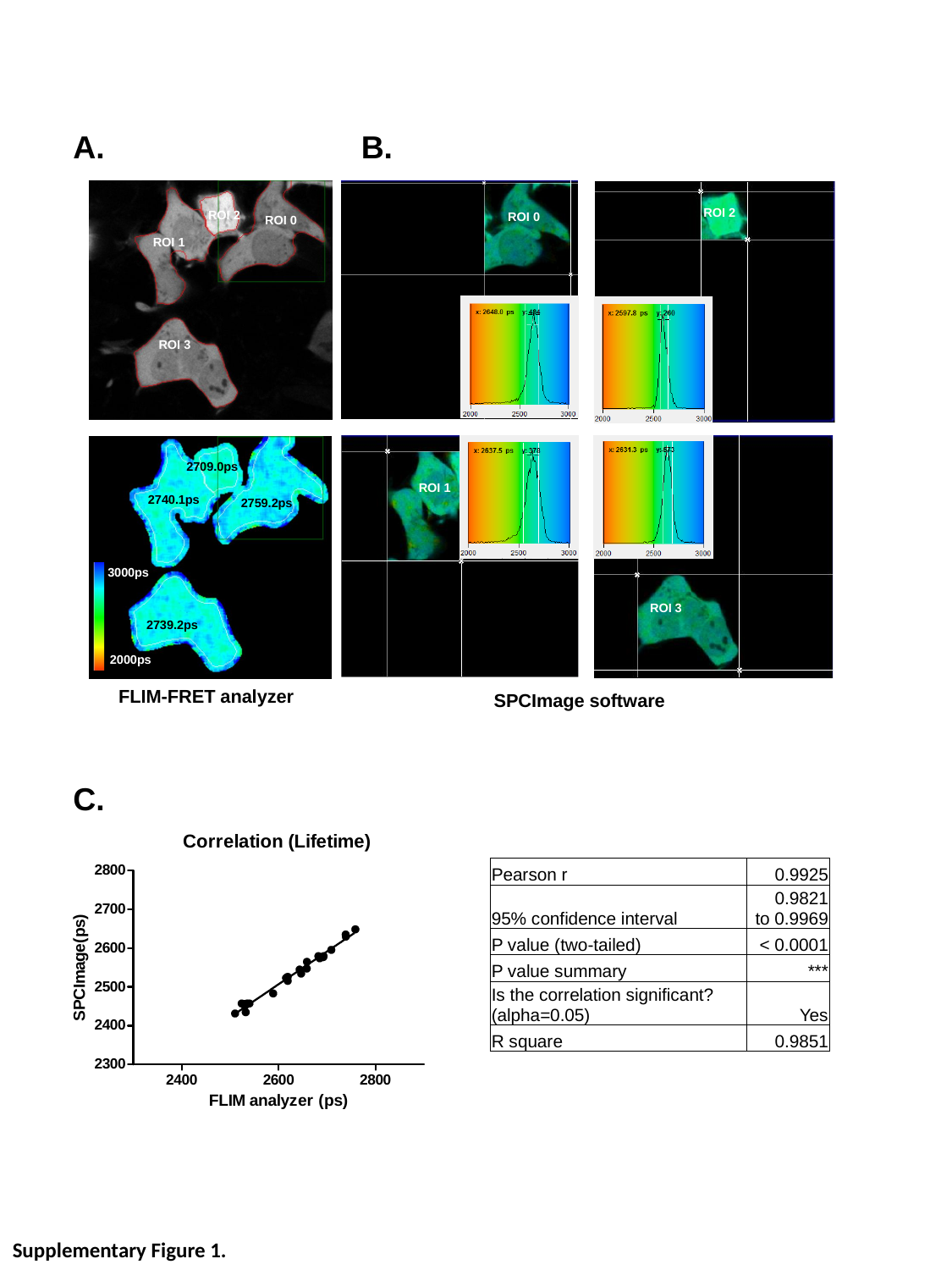

B.
A.
ROI 2
ROI 2
ROI 0
ROI 0
ROI 1
ROI 3
2709.0ps
ROI 1
2740.1ps
2759.2ps
3000ps
ROI 3
2739.2ps
2000ps
FLIM-FRET analyzer
SPCImage software
C.
| Pearson r | 0.9925 |
| --- | --- |
| 95% confidence interval | 0.9821 to 0.9969 |
| P value (two-tailed) | < 0.0001 |
| P value summary | \*\*\* |
| Is the correlation significant? (alpha=0.05) | Yes |
| R square | 0.9851 |
Supplementary Figure 1.

## Slide 2
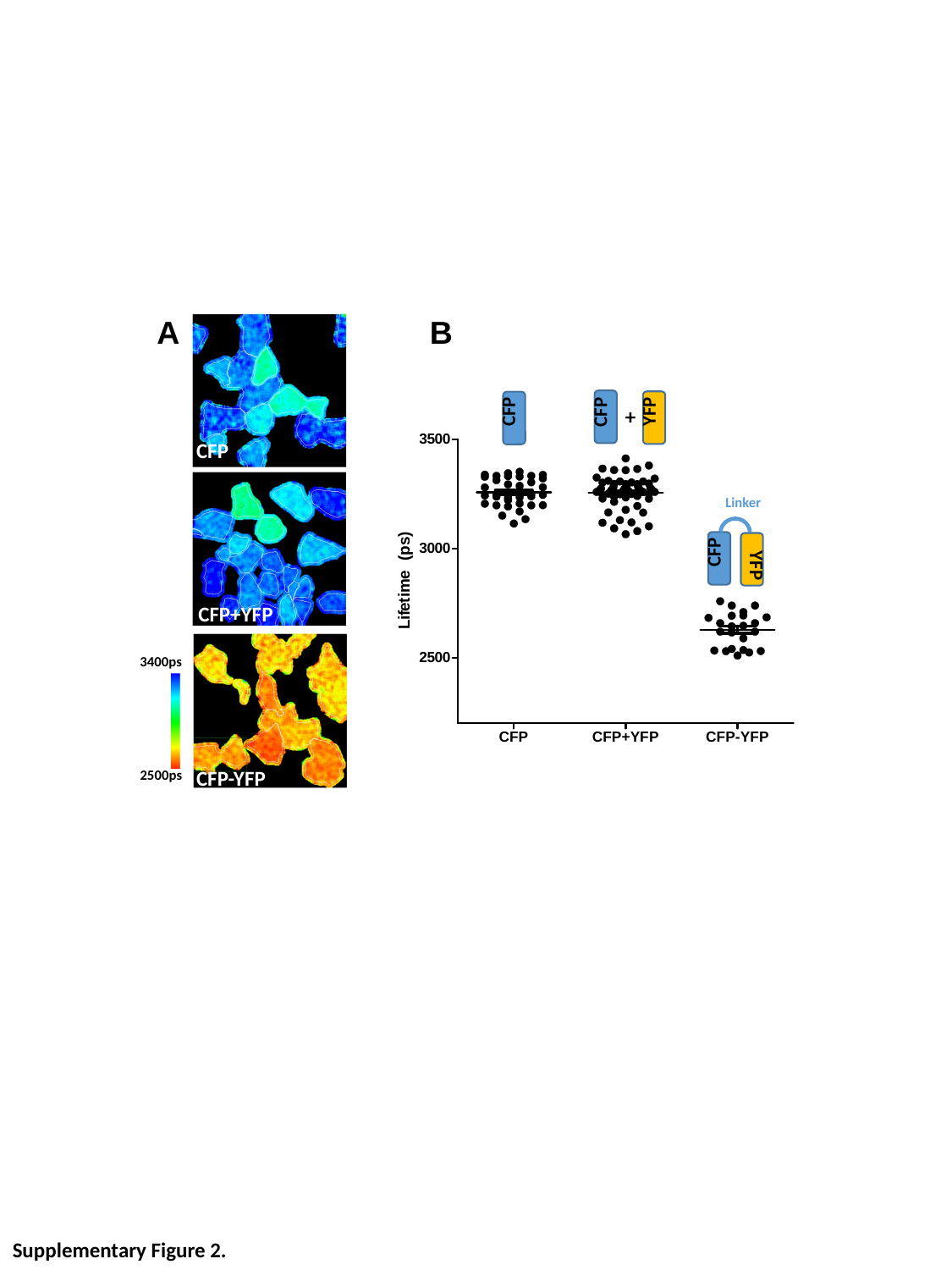

A
B
CFP
YFP
+
CFP
CFP
CFP
Linker
CFP
YFP
CFP+YFP
3400ps
CFP-YFP
2500ps
Supplementary Figure 2.
