# Supplementary material for: FLIM-FRET analyzer: open source software for automation of lifetime-based FRET analysis
Source: Source Code Biol Med. 2017 Nov 3;12:7. doi: 10.1186/s13029-017-0067-0 (PMC5670528; doi:10.1186/s13029-017-0067-0)

**
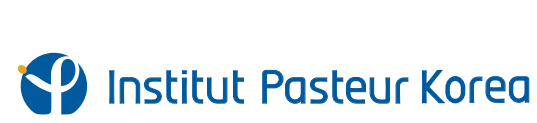
**

**FLIM-FRET Analyzer user guide**

**
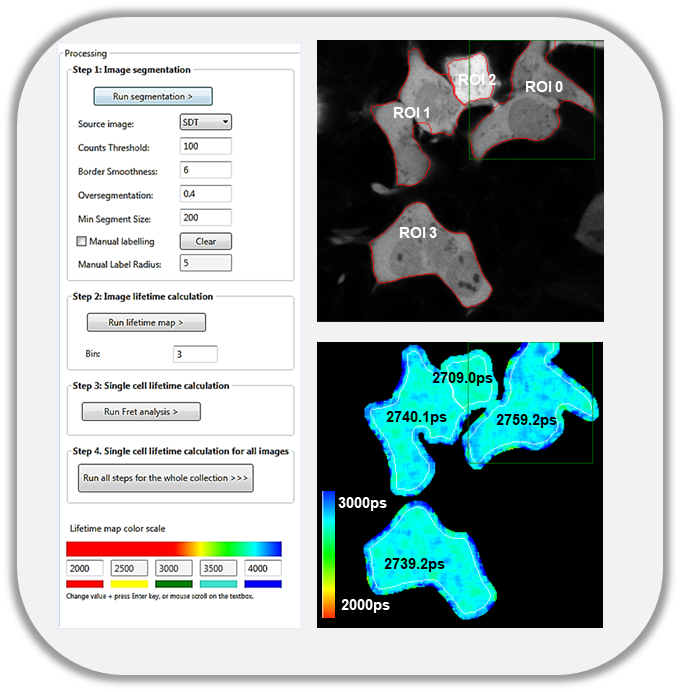
**

**Contents**

**Introduction…………………………………………………………….……….……….3**

**Basic concept……………………………………………………..……………...……….3**

**Interface overview……...…………………………………………………… ………….3**

**Step by step process**

**1-Create FRET collection…………………………………………....………….4**

**2- Open FRET collection……………………………………………...………...5**

**3- Single cell segmentation……………………………………………………...6**

**4-Image lifetime calculation…………………………………………………….8**

**5-Single cell lifetime calculation………………………………………….…….9**

**6-Bathched Single cell lifetime calculation……………..…………………….10**

**7-Bathched Single cell lifetime calculation for image collection…………….11**

**Segmentation …………………………………………………………………...………12**

**Fitting model……………………………………………………………………………13**

**Troubleshooting………………………………………………………………………...14**

**Introduction**

FLIM-FRET analyzer is an open source software is dedicated to fluorescence lifetime imaging microscopy (FLIM) data obtained from Becker & Hickl SPC-830. FLIM-FRET analyzer includes: a user-friendly interface enabling automated intensity-based segmentation into single cells, time-resolved fluorescence data fitting to lifetime value for each segmented objects, batch capability, and data representation with donor lifetime versus acceptor/donor intensity quantification as a measure of protein-protein interactions.

**Basic concept**

FLIM-FRET analyzer is an open source software: FLIM-FRET analyzer is public domain open source software. You are free to run, redistribute or improve the program so that the whole community benefits.

**Interface overview**

**
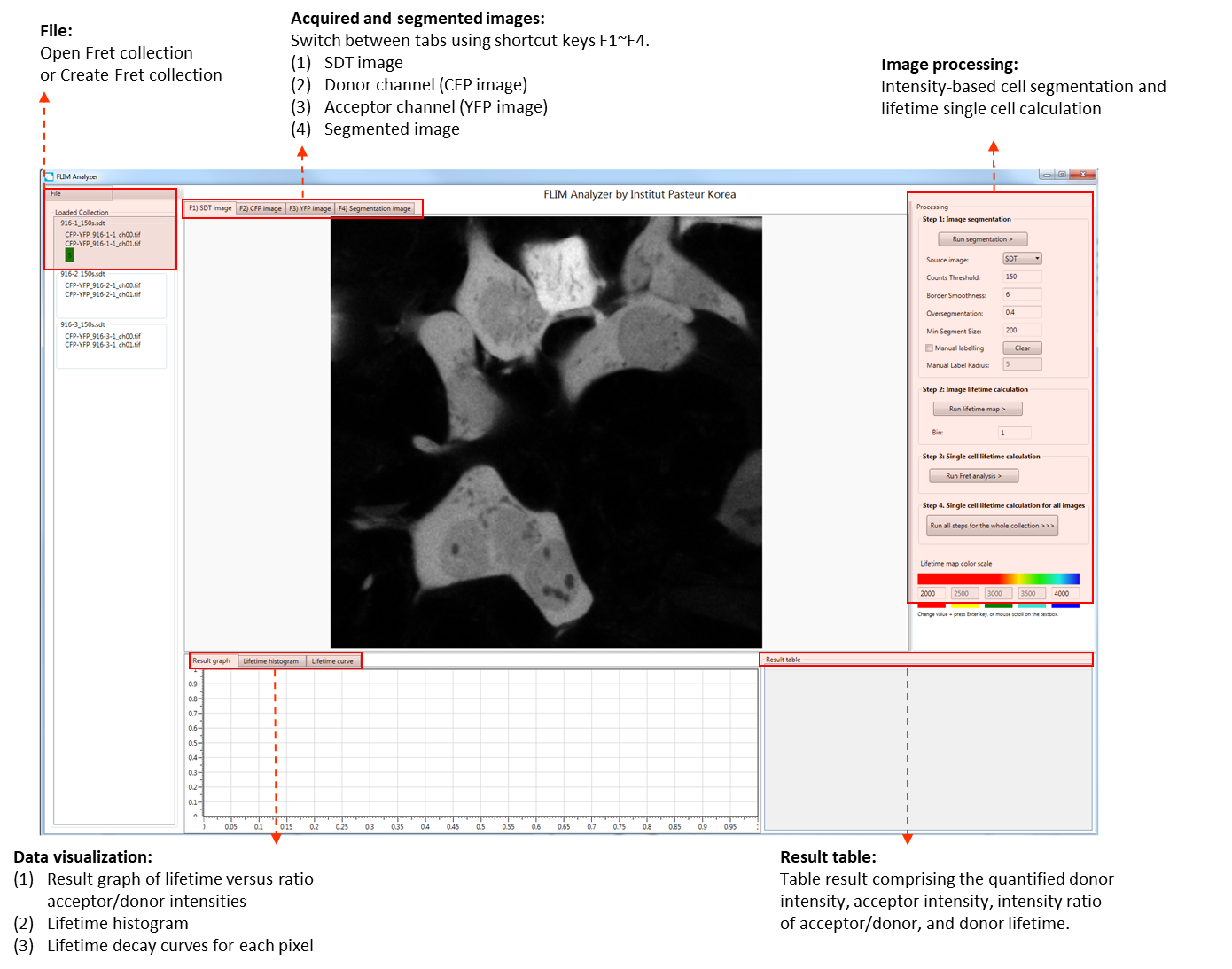
Step by step process**

**1-Create FRET Collection**

In order to create the FRET collection, organize your acquisition data corresponding to the fluorescence (TIF files) and lifetime (SDT files).


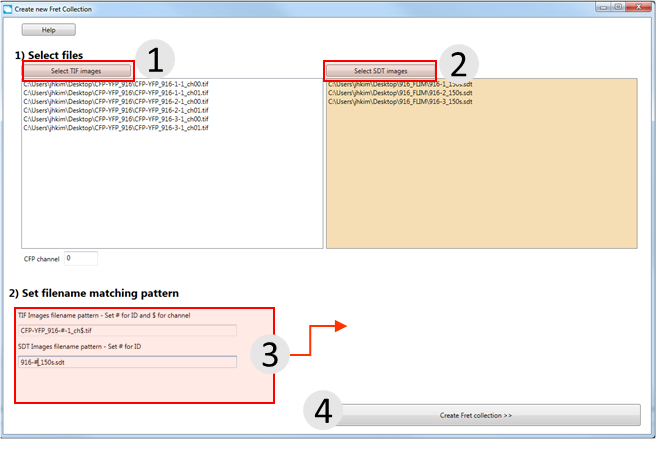


- 1. As refer to the figure above, select at position 1 the TIF images that correspond to the donor (file name ending ch00) and acceptor (file name ending 01) fluorescent channels,
  2. Position 2, select the FLIM file from the donor (SDT file extension is associated with the SPCImage software).
  3. Position 3, set file name matching pattern using # for experiment ID and $ for the fluorescence channel.
  4. Position 4, press the icon ‘Create Fret Collection’ and a new window will appear as bellow seen bellow.


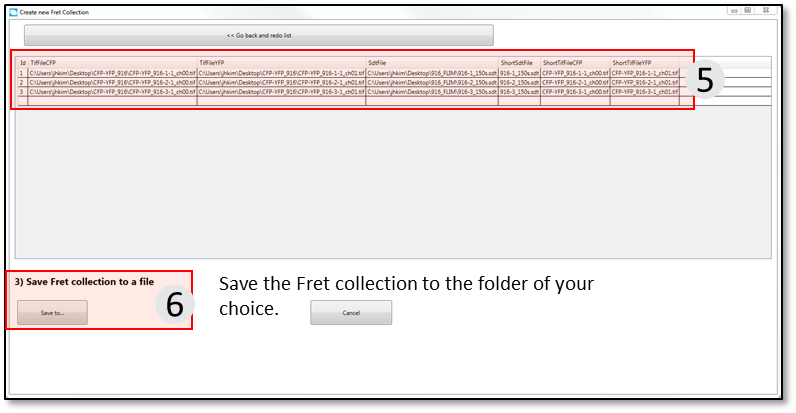


- 1. Position 5, the new collection is generated.
  2. Position 6, press the icon ‘Save Fret collection to a file’ to save the current collection on your computer or server for further study.

**2- Open FRET Collection**


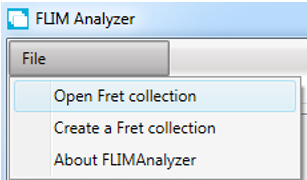


2.1 Open the previously saved data upon selection in the File menu the Open Fret collection.


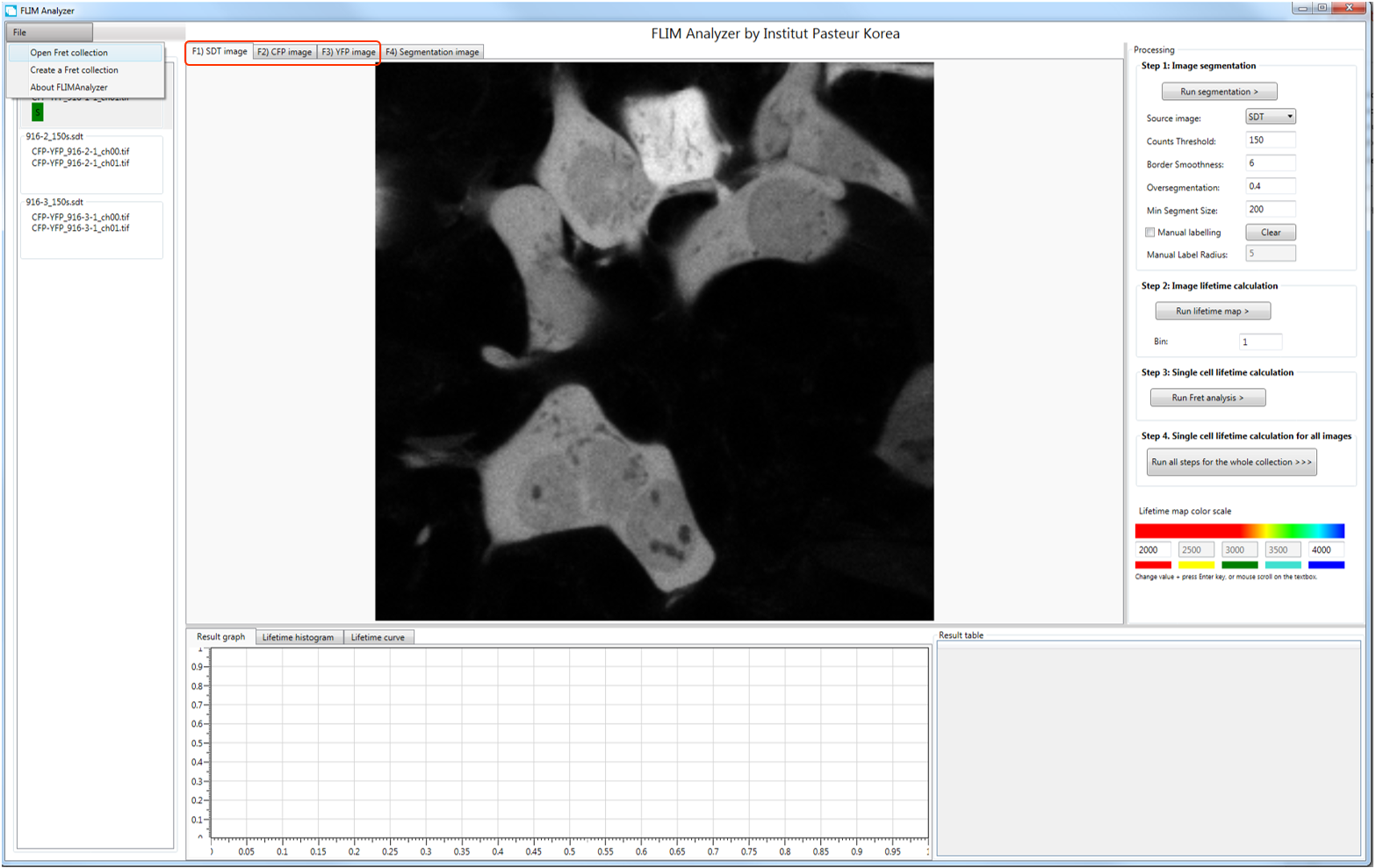


Below shows a magnified element of FLIM-FRET analyzer.


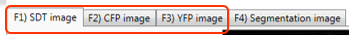


2.2 After loading the file, you can see the F1)SDT image, F2)CFP image, F3)YFP image on the different tabs.

**3- Single Cell Segmentation (Step 1)**

**
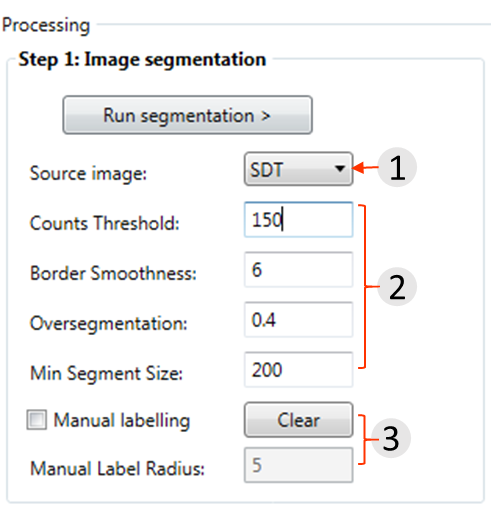
**

**3 .1** Select the source image for the segmentation of cell of interest. (You can select any of loaded images in your FRET collection (CFP, YFP, or SDT image)

**3.2** **Automated segmentation**: Set the segmented cell characteristics based on multiple parameters as below.

- **Counts Threshold** – minimal number of photons necessary to register a signal, in order to separate background from foreground
- **Border Smoothness** – parameter that controls trade-off between smoothness and number of details of the segments’ borders. Greater values lead to more smooth borders.
- **Over-segmentation** – parameter that indirectly controls number of produced segments. Should be between 0 and 1. Bigger values correspond to larger number of produced segments. When the parameter is 1 it is likely that the image is over-segmented.
- **Min Segment Size** – defines minimal size of a segment, necessary to keep this segment.

**3.3** **Semi-automated segmentation**

- **Manual labelling** is available to assist in the segmentation. Enabled by clicking on the respective check-box. In this mode user is asked to click on the centers of the cells. The locations are marked by green squares and are used as seed points during the segmentation.
- **Manual Label Radius** – defines size of manually placed markers and controls shapes of resulting segments (the resulting segments will contain an entire marker).


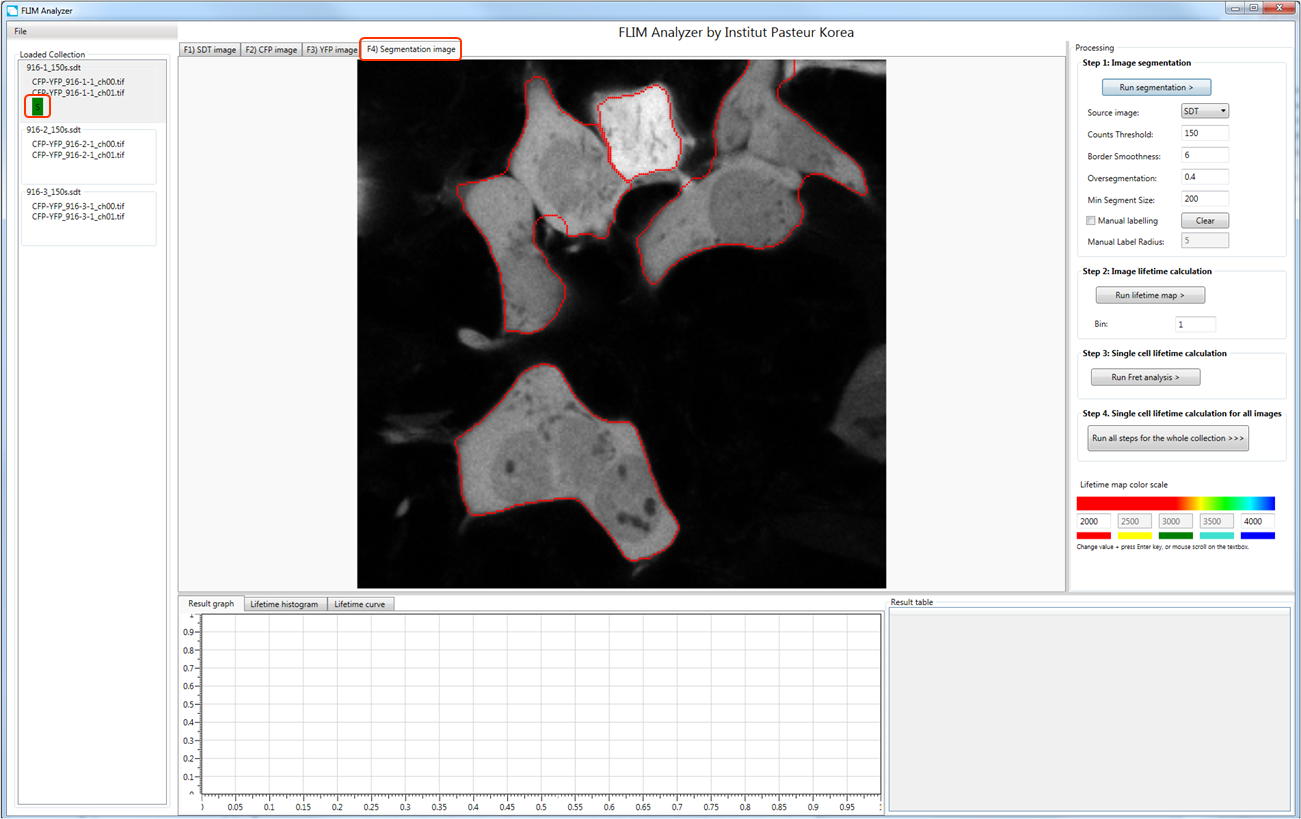


Below shows a magnified element of FLIM-FRET analyzer.


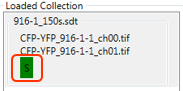

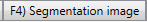


-The green **“S” icon** means that the ‘Image segmentation’ was previously performed

-After performing ‘Image segmentation’ step, we can see the segmentation image by clicking the **“F4) Segmentation image”** tab.

**4-Image lifetime calculation (Step 2)**

- 1. Set the binning value. Increasing binning value allows better lifetime data fitting at the expense of the FLIM image resolution. 🡺 Click on the ‘Run lifetime’ map icon to launch the image lifetime calculation.
  2.
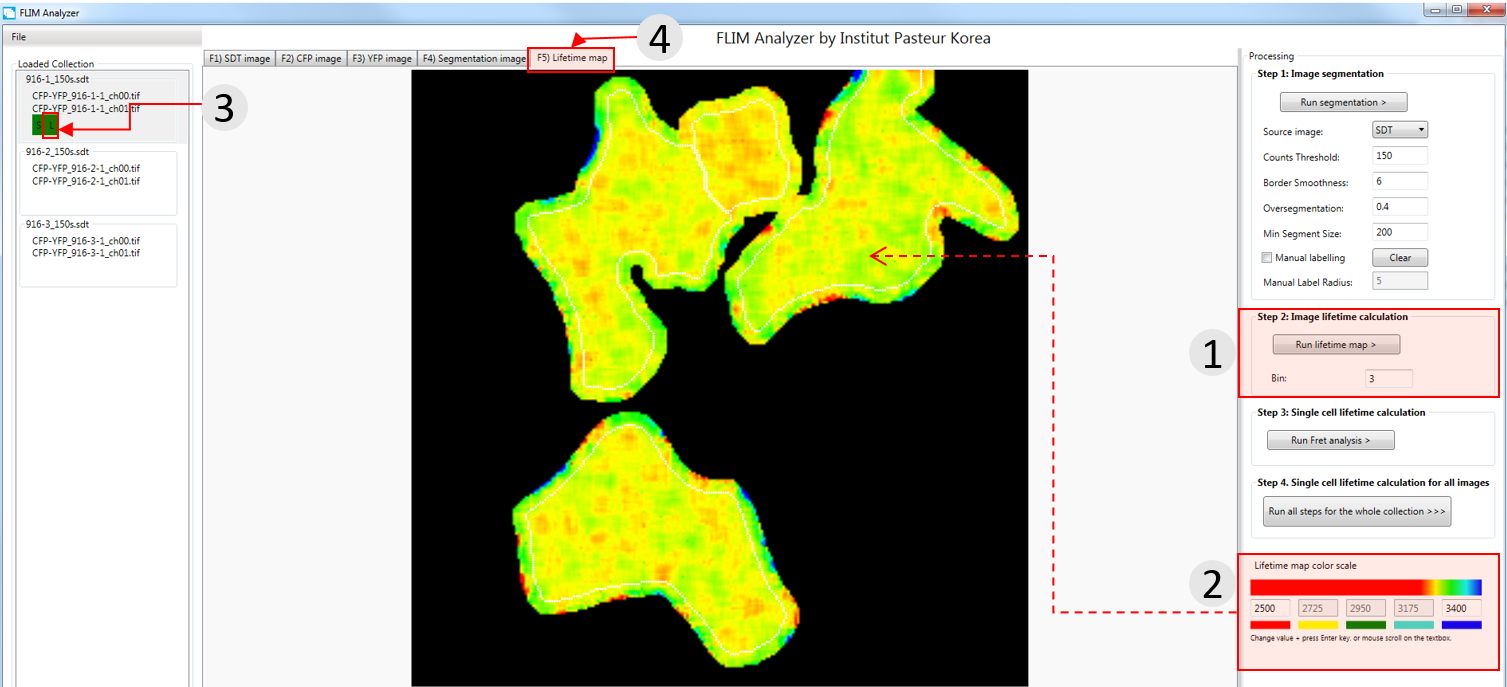
Upon ‘Image lifetime calculation’ step, a lifetime image based on the lifetime map color scale is displayed. Red color represents shorter lifetime and blue color represents longer lifetime.
  3. This **“L” icon** shows that the “Image lifetime calculation” was previously performed.
  4. After performing “Image lifetime calculation” step, we can see the lifetime image by clicking the **“F5) Lifetime map”** tab.
  5.
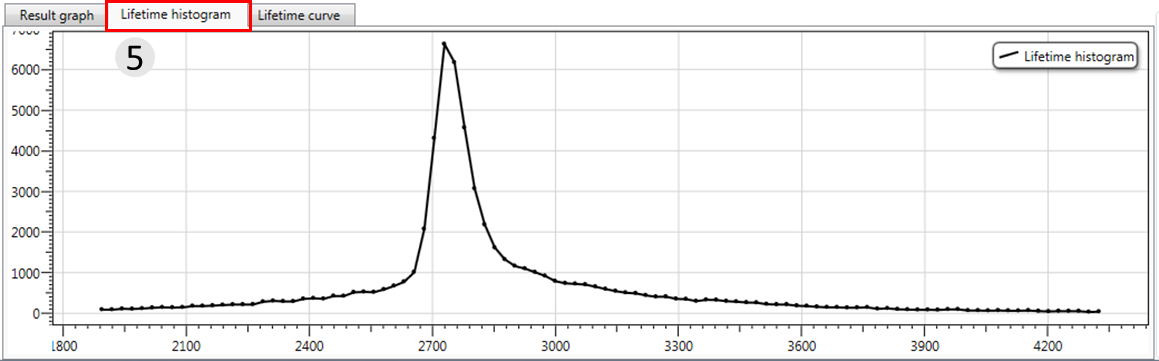
 **“Lifetime histogram” tab** shows the **Fluorescence lifetime distribution** for the selected image.

**4.6** **“Lifetime curve” tab** shows **the fluorescence lifetime decay curves** of the image area pointed with the mouse cursor at SDT image.

**
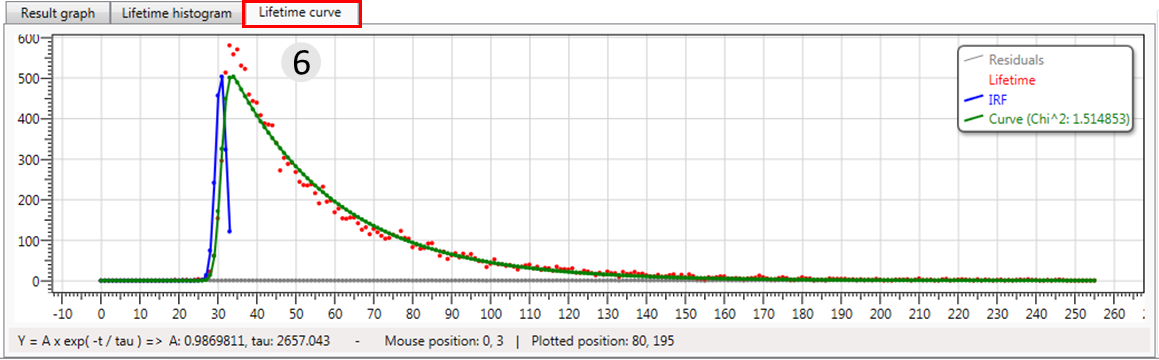
**

**5-Single cell lifetime calculation (Step3)**

**5.1** Upon clicking **“Run Fret analysis”,** the FLIM and fluorescence image data are processed and are calculated the fluorescence intensity of the donor (CFP) and acceptor (YFP), the fluorescence ratio (YFP/CFP) and the lifetime values for every segmented cell.

**5.2** This **“R” icon** shows that the “Single cell lifetime calculation” was previously performed.

**5.3** Single cell lifetime calculation data were listed in **“Result table”**. The process data for the selected cell framed in a green rectangle appears as well highlighted in grey in the result table.

**
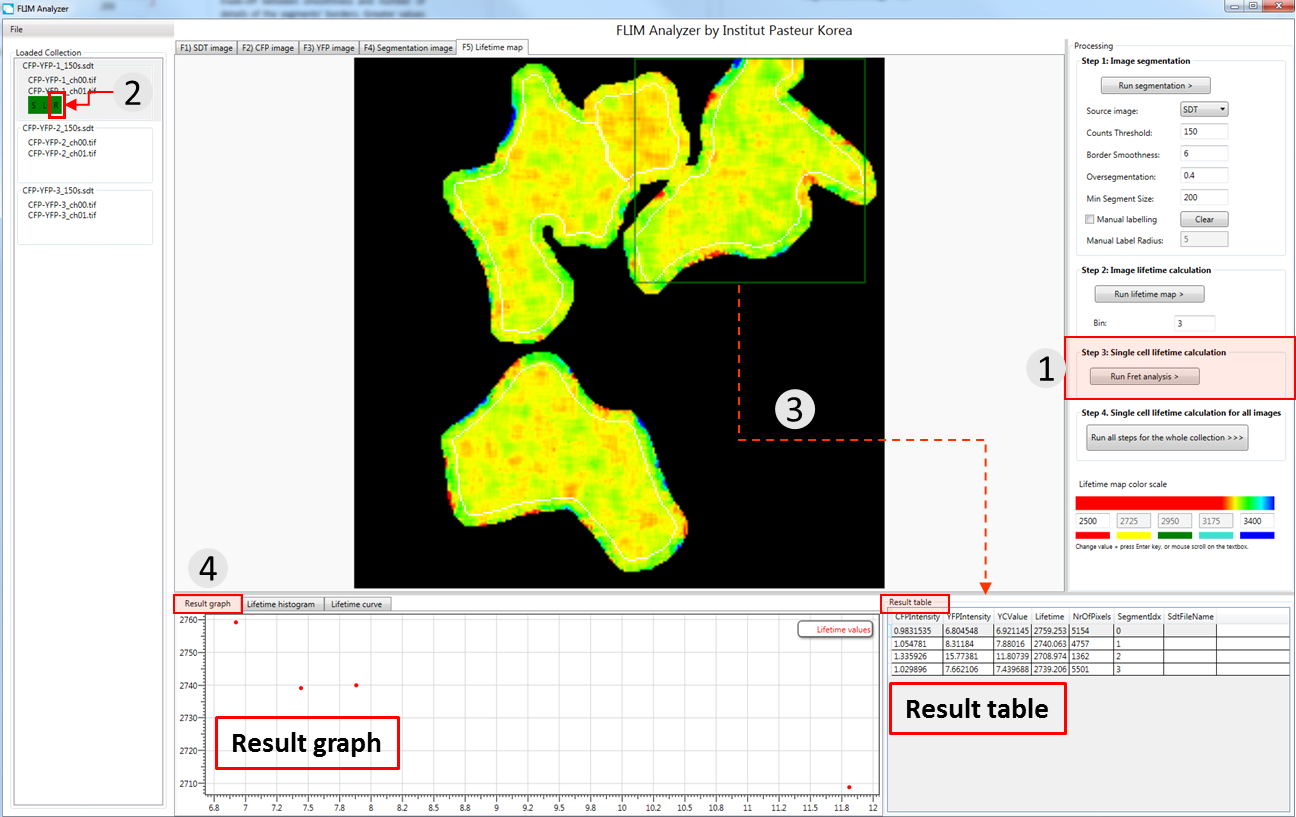
5.4** Single cell lifetime calculation data were also represented in **“Result graph”** as Y/C ratio vs. fluorescence lifetime.

**6-Bathched Single cell lifetime calculation (Step 4: Single cell lifetime calculation for all images)**

**6.1** Upon clicking the icon **“Run all steps for the whole collection”**, whole fret data will be prepared for batch process.


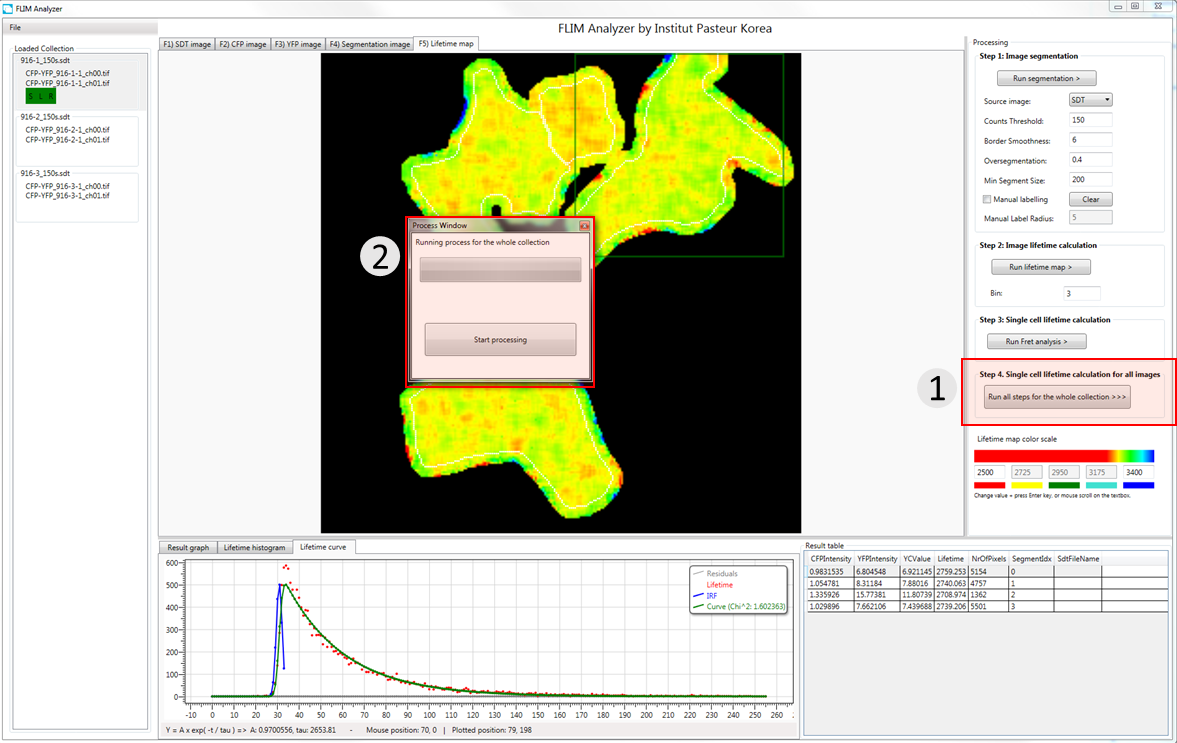
**6.2** Click on the icon **“Start processing”** to launch the batch processing of the Fret collection.

**7-Bathched Single cell lifetime calculation for image collection**


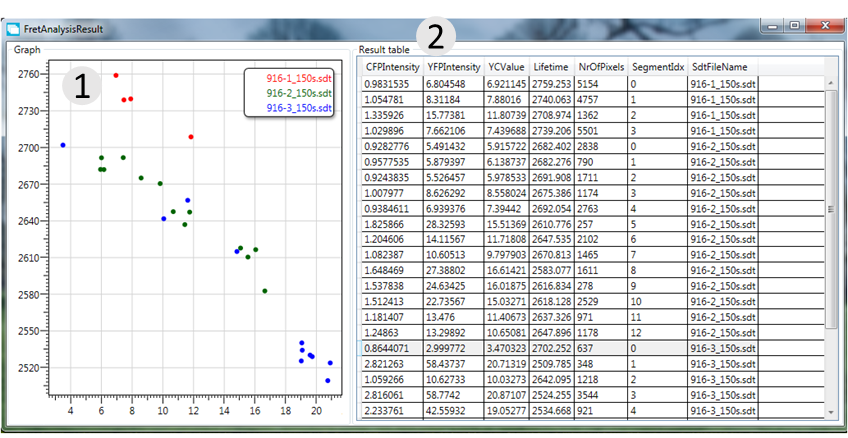
After completing the analysis for each single cell, the analysis results are displayed in a pop-up window **(FretAnalysisResult: Graph+Result table)** as below.

**7.1** **Graph** panel shows the correlation between YFP/CFP intensity ratio and fluorescence lifetime (ps). 🡺 Different data set were represented as different color dots.

**7.2** **Result table** shows the batched single cell lifetime analysis results for each single cell.

- **CFP intensity**: Average fluorescence intensity of the donor fluorophore (CFP) measured for each single segmented cells.
- **YFP intensity**: Average fluorescence intensity of the acceptor fluorophore (YFP) measured for each single segmented cells.
- **YCValue**: Ratio of YFP intensity/CFP intensity.
- **Lifetime**: Average lifetime of the donor measured for each single segmented cells.
- **NrOfPixels**: total count of pixel measured for each segmented cells.
- **SegmentIdx**: ID for each cell within the one image.
- **SdtFileName**: file name for each lifetime image

**Segmentation**

Segmentation parameter can be tuned to adapt to low and high cell density as seen in the two examples bellow.

Example-1:


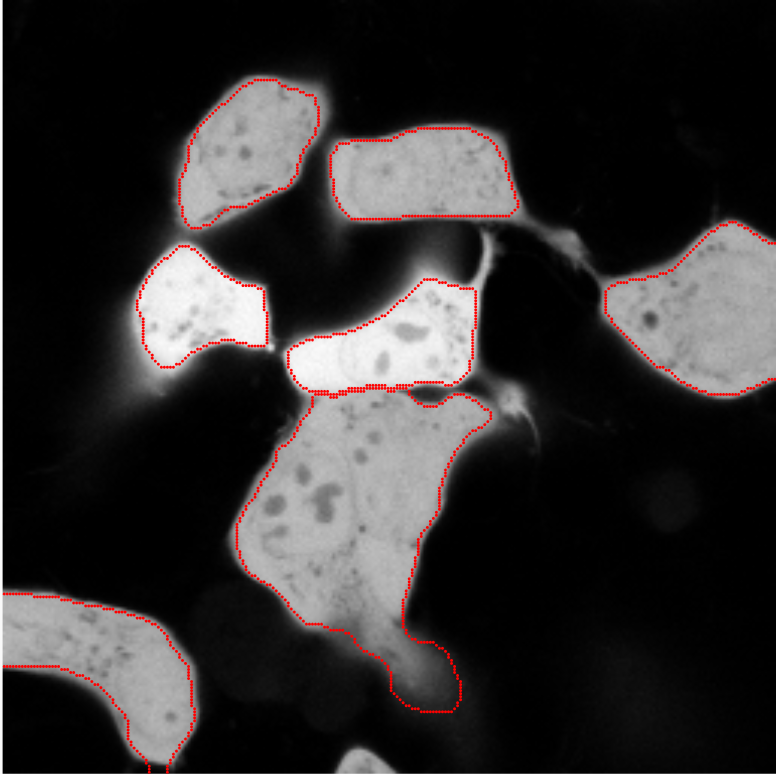

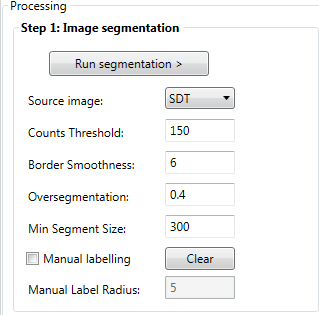


Example-2:


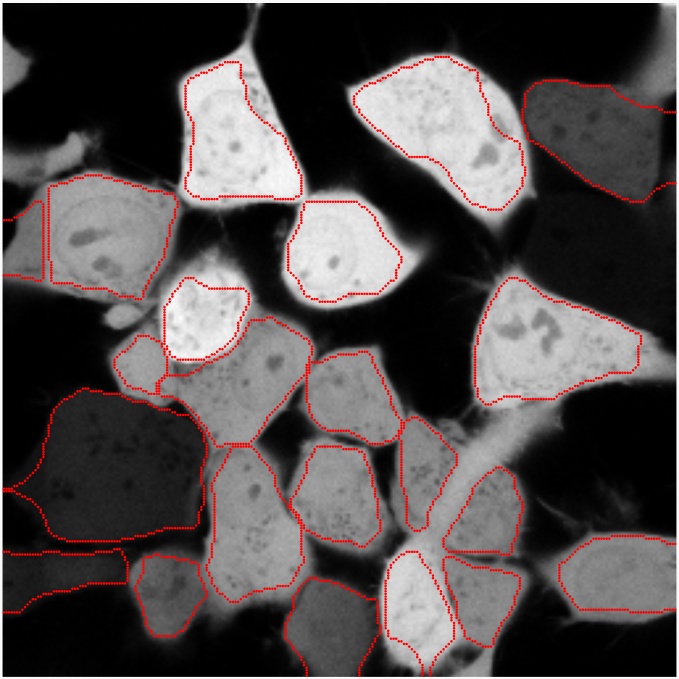

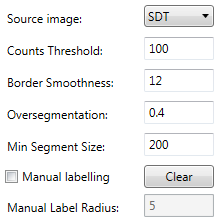


**Fitting model**

FLIM-FRET analyzer propose a single exponential decay function. The following exponentially modified Gaussian was used.


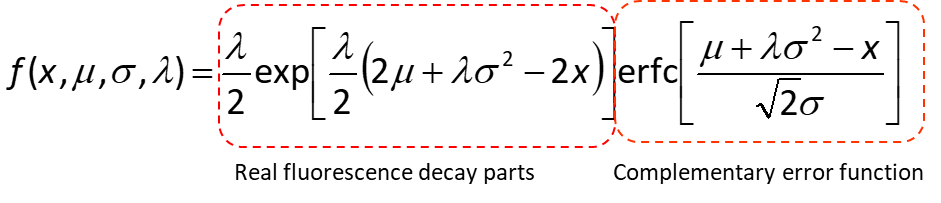


1-Argument: x

2- Optimization parameters: μ,σ,λ, which control mean, spread and rate of decay of the resulting curve respectively: μ corresponds to the location of peak, σ controls its sharpness (the smaller it is, the more sharp the peak becomes), and λ is proportional to rate of 'decay' of the right tail.

2-Complementary error function: erfc

Fitting example as bellow.


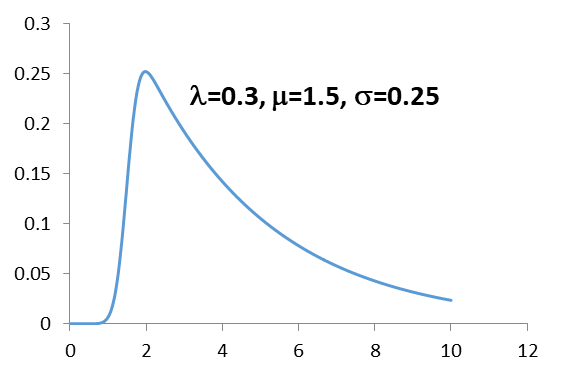


**Troubleshooting**

**1-**It is important to use the FLIM-FRET analyzer together with a so called “fret collection”, which specifies how tiff and sdt files are associated. Such collection, should be created and saved first prior any use of FLIM analysis software. The step are now further explained in the new ‘FLIM-FRET analyzer manual’ as:

(1) Select “File Create a Fret collection” to create a collection description and specify its filename;

(2) Once the collection is saved, open the collection “File Open Fret collection” in the FLIM-FRET analyzer to do the analysis.

**2-**We recommend the user to check prior usage of FLIM-FRET analyzer that MS Windows .NET Framework 4.0 (https://www.microsoft.com/en-us/download/details.aspx?id=17851) is installed.

**3-**In case of troublesome for those using an upper version operating system than Windows 7, such as Windows 8 or Windows 10, a potential solution would be to run FLIM-FRET analyzer in the “Compatibility mode”. This can be enabled by right-click on the executable file (FLIM-Analyzer.exe), and selecting “Properties”. This should open the dialog window, with the tab “Compatibility”. In this tab check the box in the “Compatibility mode” group and select “Windows 7” in the drop-down menu.


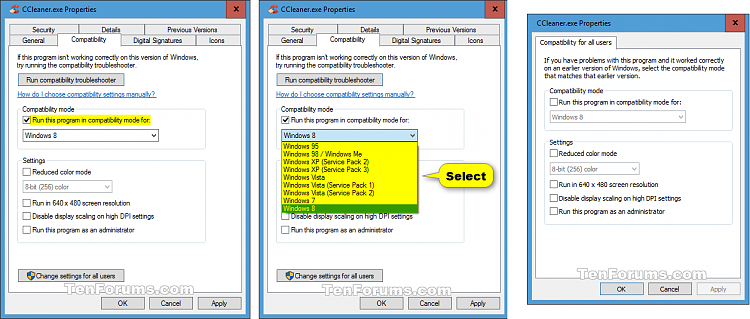

Supplement: Supplementary file 2 — User guide. (DOCX 4536 kb) [file 13029_2017_67_MOESM2_ESM.docx]
